# Acute Pancreatitis Secondary to Doxycycline, a Widely Prescribed Antibiotic: A Case Report

**DOI:** 10.7759/cureus.84115

**Published:** 2025-05-14

**Authors:** Gloria Erazo Montalvan, Guy Loic Nguefang Tchoukeu, Niempa Bacani, Kelash Bajaj

**Affiliations:** 1 Internal Medicine, Texas Tech University Health Sciences Center, Odessa, USA; 2 Hematology and Oncology, Texas Tech University Health Sciences Center, Odessa, USA

**Keywords:** acute pancreatitis, adverse drug reaction, antibiotic, doxycycline, drug-induced pancreatitis

## Abstract

Acute pancreatitis is a leading cause of hospital admissions in the United States. Although drug-induced acute pancreatitis is considered rare, it is an important etiology to consider. We report a case of acute pancreatitis potentially due to doxycycline. A 59-year-old woman with a history of breast cancer, undergoing chemotherapy with palbociclib and fulvestrant for the last two years, was admitted for severe epigastric pain radiating to the back. Six days earlier, she had started doxycycline for an upper respiratory infection. Serum lipase was 837 U/L, a computed tomography scan showed diffuse acute pancreatitis without necrosis, and common etiologies such as gallstones, alcohol use, and metabolic disorders were excluded. On admission, doxycycline was discontinued, and the patient reported significant improvement in symptoms with supportive care within four days. Because of the temporal association and exclusion of other causes, doxycycline was identified as the most likely etiology. By presenting this case, we want to highlight the importance of considering doxycycline as a potential cause of acute pancreatitis and underscore the need for heightened doxycycline pharmacovigilance to improve patient safety.

## Introduction

Acute pancreatitis (AP) is one of the leading causes of hospitalization among gastrointestinal disorders worldwide [1]. The global incidence of AP has been steadily rising in most countries, with North America experiencing a rise from 2.76% in 1961 to 4.57% in 2016 [2]. In the United States, the diagnosis of AP accounts for an estimated 230,000-275,000 hospitalizations annually, with mortality ranging from 3% in mild cases to 20% in severe cases [1,3]. The most frequent etiologies include gallstones, alcohol, hypercalcemia, hyperlipidemia, post-endoscopic retrograde cholangiopancreatography (post-ERCP), infections, and trauma [2,4]. Drug-induced pancreatitis (DIP) is rare, comprising 0.1-2% of AP cases, and its pathophysiology remains poorly understood at this time [5]. Among potential drug etiologies, doxycycline, part of the tetracycline class, has been implicated in only some case reports [5,6]. Given the global use of doxycycline and its potential to cause severe adverse effects, it is important for clinicians to recognize this rare complication. We describe a case of doxycycline-induced pancreatitis, intending to raise the awareness of clinicians and contribute to the pharmacovigilance of this commonly prescribed medication.

## Case presentation

A 59-year-old woman with a medical history of metastatic breast cancer was previously treated with a left mastectomy and is currently undergoing chemotherapy with palbociclib and fulvestrant for two years. She presented to the emergency department with a sudden onset of severe epigastric pain radiating to her back. The pain started three hours earlier and was sharp, progressive, and associated with multiple episodes of non-bloody emesis. She denied any history of pancreatitis, alcohol use, or gallstones, smoking, fever, trauma, recent procedure, or recent weight loss. Six days before her admission, she started doxycycline 100 mg daily for the treatment of an upper respiratory infection with improvement.

**Figure 1:**
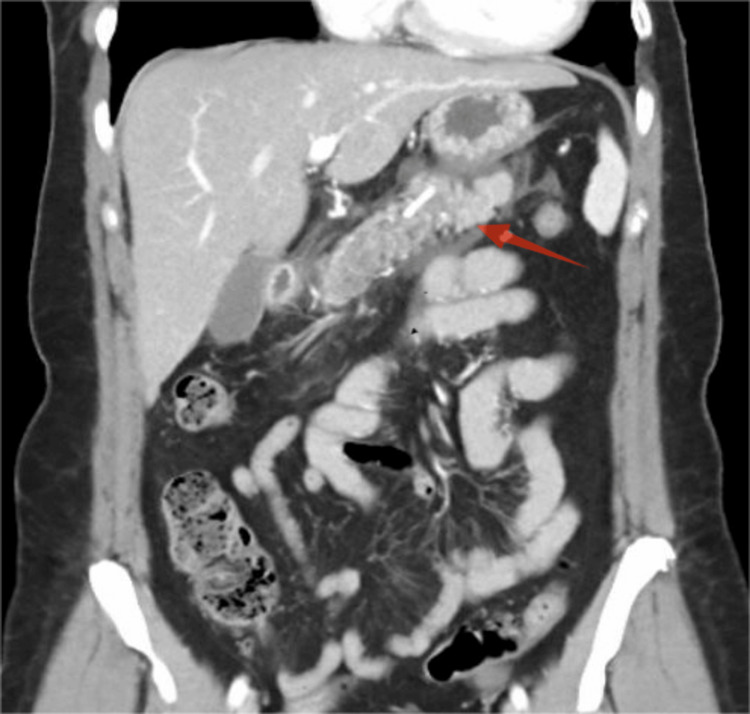
Contrast-enhanced CT of the abdomen and pelvis revealing diffuse acute pancreatitis without necrosis CT: computed tomography

**Figure 2:**
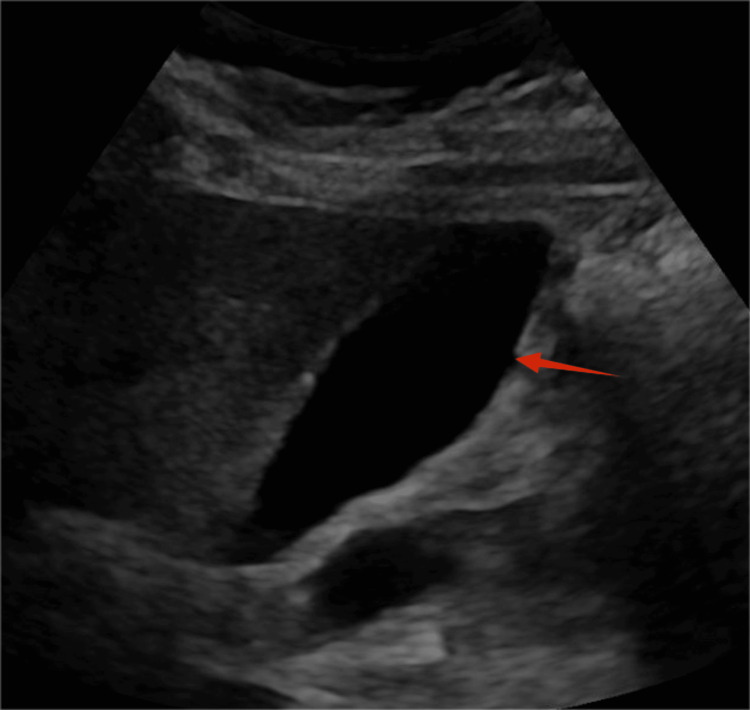
Right upper quadrant ultrasound revealing a normal-appearing gallbladder or absence of gallstones or wall thickening

On admission, she was hemodynamically stable. Physical examination was significant for epigastric tenderness, with hypoactive bowel sounds. No rebound tenderness or abdominal wall rigidity was noted. Initial laboratory studies were remarkable for serum lipase of 837 IU/L, more than three times the upper limit of normal, lactate dehydrogenase (LDH) of 243, and leukopenia of 2.6×10^3^/mcL. Serum calcium, lipid panel, renal function, and liver enzyme levels were within normal range. Urinalysis was notable for ketonuria (40 mg/dL) and glucosuria (70 mg/dL). The ethanol level was negative. Given the recent respiratory infection and leukopenia, a respiratory panel was ordered to rule out an active infection, but the results were negative. Viral infections, such as COVID-19 and hepatitis B and C, were negative (Table 1). Unfortunately, we did not test the patient for echovirus or coxsackievirus. The leukopenia was then attributed to chemotherapy and was temporarily discontinued during her hospital stay. A contrast-enhanced computed tomography (CT) scan of the abdomen showed diffuse AP without necrosis (Figure 1). A right upper quadrant ultrasound revealed a normal-appearing gallbladder and biliary tree (Figure 2). Supportive care was initiated, including IV Ringer's lactate for hydration, ondansetron for nausea and vomiting, multimodal pain management with acetaminophen, hydrocodone, and morphine, deep vein thrombosis prophylaxis with enoxaparin, and a liquid diet. Because all the well-known causes of pancreatitis were ruled out, doxycycline was the only medication that could explain the presentation and was discontinued. After four days, the patient reported improvement in her epigastric pain. Her diet was advanced as tolerated, and she was discharged in stable condition after six days.

**Table 1:** Laboratory results on admission The table was created by the authors based on the information from the patient's chart. WBC: white blood cell; RBC: red blood cell; HDL: high-density lipoprotein; BUN: blood urea nitrogen; AST: aspartate transferase; ALT: alanine transaminase; ALP: alkaline phosphatase; LDH: lactate dehydrogenase; RSV: respiratory syncytial virus

Test	Result	Reference range
Complete blood count		
WBC	2600	4800-10800 cells/mcL
RBC	3.30	4.20-5.40 million cells/mcL
Hemoglobin	12.1	12.0-16.0 g/dL
Hematocrit	36	37-47%
Platelets	189000	130000-400000 cells/mcL
Lipid panel		
Cholesterol	191	≤200 mg/dL
Triglycerides	123	≤150 mg/dL
HDL	50	≥60 mg/dL
Metabolic panel		
BUN	20	6-20 mg/dL
Creatinine	0.8	0.5-0.9 mg/dL
Sodium	143	136-145 mmol/L
Potassium	3.5	3.5-5.1 mmol/L
Calcium	9.3	8.6-10 mg/dL
Protein total	6.9	6.4-8.3 g/dL
Albumin	4.2	3.5-5.2 g/dL
AST	24	≤35 unit/L
ALT	18	≤35 unit/L
ALP	72	35-104 unit/L
LDH	243	135-214 unit/L
Lipase	837	13-60 unit/L
Ethanol level	0	100-200 mg/dL
Urine analysis		
UA color	Light yellow	-
UA glucose	70	0 mg/dL
UA ketones	40	0 mg/dL
UA-specific gravity	1.048	1.003-1.040
UA pH	5.5	5.5-6.5
Other		
Respiratory panel tested for adenovirus, coronavirus, metapneumovirus, rhinovirus, influenza A and B, parainfluenza, RSV, *Bordetella pertussis*, *Chlamydia pneumoniae*, and *Mycoplasma pneumoniae*	Not detected	Not detected

## Discussion

The prevalence of DIP is very low, ranging between 0.1% and 2% of all AP cases [5]. The pathophysiologic mechanisms are poorly understood and are mainly based on theories. Among tetracyclines, drugs like minocycline, tigecycline, and demeclocycline have been more commonly associated with AP, whereas doxycycline is rarely implicated [5]. AP has been reported in less than 1% of patients taking doxycycline, but interestingly, several case reports in the literature have identified doxycycline as a potential etiology of AP [4,7-9].

Our patient met the criteria of the Revised Atlanta classification of AP [10], presenting epigastric pain, elevated lipase levels exceeding three times the upper limit of normal, and contrast CT of the abdomen showing diffuse AP without necrosis. Common causes, such as alcohol, gallstone, hypercalcemia, hypertriglyceridemia, post-ERCP pancreatitis, and infection, were excluded based on the history, physical examination, and laboratory findings. The only potential etiology was the medications started before admission. In this case, the patient was on chemotherapy with palbociclib and fulvestrant and doxycycline for an upper respiratory infection before admission. Chemotherapy drugs were not incriminated because the patient had been on these medications for over two years without any issues. Further, we did not find evidence in the literature suggesting that these drugs could cause AP. However, doxycycline was the only medication started a few days before the onset of symptoms, making it the most likely cause. Regardless, both the chemotherapy drugs and doxycycline were discontinued on admission, respectively, because of leucopenia and AP. The patient's symptoms improved, supporting the idea that doxycycline was the cause of her pancreatitis.

The diagnosis of doxycycline-induced pancreatitis relies mostly on the physician's ability to systematically rule out other potential causes while considering the comorbidities and concomitant medications that may contribute to the condition [11-13]. The best way to establish doxycycline as the causative agent is through a rechallenge test, but this approach violates ethical norms. Interestingly, Bassi et al. documented a case of recurrent pancreatitis attributed to doxycycline in a patient with a history of a similar episode two years earlier, providing indirect evidence of causation through an unintentional rechallenge test [4].

The Naranjo Adverse Drug Reaction Probability Scale, used to assess causality in cases of adverse drug reactions, yielded a score of 6 (Table 2) in our case, indicating doxycycline as a probable cause of AP [14]. In contrast, in the case report by Kakes et al., the Naranjo score was 4, indicating an association between doxycycline and the onset of AP [12]. Based on the descriptions in other cases [4,7,9], we can subjectively assign a Naranjo score ranging between 4 and 8, providing further evidence of doxycycline-induced pancreatitis. Additional research could help in understanding the pathophysiology and the true incidence of this rare adverse drug reaction. Such studies would help in improving pharmacovigilance, identifying populations at higher risk, and improving overall patient safety.

**Table 2:** Patient's calculated Naranjo score The table was created by the authors based on the information from the Naranjo Adverse Drug Reaction Probability Scale [14]. Interpretation: Doubtful ADR (<2): The reaction was likely related to factors other than a drug. Possible ADR (2-4): The reaction followed a temporal sequence after a drug, possibly followed a recognized pattern to the suspected drug, and could be explained by characteristics of the patient's disease. Probable ADR (5-8): The reaction followed a reasonable temporal sequence after a drug, followed a recognized response to the suspected drug, was confirmed by withdrawal but not by exposure to the drug, and could not be reasonably explained by the known characteristics of the patient's clinical state. Definite ADR (≥9): The reaction followed a reasonable temporal sequence after a drug or in which a toxic drug level had been established in body fluids or tissues, followed a recognized response to the suspected drug, and was confirmed by improvement on withdrawing the drug and reappeared on reexposure. ADR: adverse drug reaction

Question	Yes	No	Don't know	Patient's score
1. Are there previous conclusive reports on this reaction?	+1	0	0	+1
2. Did the adverse event appear after the suspected drug was given?	+2	-1	0	+2
3. Did the adverse reaction improve when the drug was discontinued or a specific antagonist was given?	+1	0	0	+1
4. Did the adverse reaction appear when the drug was readministered?	+2	-1	0	0
5. Are there alternative causes that could have caused the reaction?	-1	+2	0	+2
6. Did the reaction reappear when a placebo was given?	-1	+1	0	0
7. Was the drug detected in any body fluid in toxic concentrations?	+1	0	0	0
8. Was the reaction more severe when the dose was increased or less severe when the dose was decreased?	+1	0	0	0
9. Did the patient have a similar reaction to the same or similar drugs in any previous exposure?	+1	0	0	0
10. Was the adverse event confirmed by any objective evidence?	+1	0	0	0
Total				6

## Conclusions

DIP is a diagnosis of exclusion, requiring the systematic elimination of more common etiologies and the careful evaluation of medication histories. This patient was admitted for AP shortly after initiating doxycycline therapy without gallstones, alcohol use, or metabolic disorders. The resolution of symptoms following the discontinuation of doxycycline strongly supports its role as the causative agent. Clinical awareness of this potential adverse effect is essential to improving diagnostics and patient outcomes. This case contributes to the growing body of evidence on doxycycline-induced AP. Further research is needed to better understand the pathophysiologic mechanism and major risk factors of doxycycline-induced AP.

## References

[1] Gapp J, Tariq A, Chandra S (2024). Acute pancreatitis. StatPearls [Internet].

[2] Iannuzzi JP, King JA, Leong JH (2022). Global incidence of acute pancreatitis is increasing over time: a systematic review and meta-analysis. Gastroenterology.

[3] Chadalavada P, Simons-Linares CR, Chahal P (2020). Drug-induced acute pancreatitis: prevalence, causative agents, and outcomes. Pancreatology.

[4] Bassi R, Prakash P, Balakrishnan E, Cockey G (2022). Blame it on the drug: a rare case of recurrent doxycycline-induced pancreatitis. Cureus.

[5] Jones MR, Hall OM, Kaye AM, Kaye AD (2015). Drug-induced acute pancreatitis: a review. Ochsner J.

[6] Xiong CY, Yang YM, Zhou Y, He TS, Luo XW, Wang J, Mao CX (2024). Systematic analysis of the adverse effects of commonly used clinical tetracycline drugs based on the FAERS database. Expert Opin Drug Saf.

[7] Vanerio P, Pontillo M, Morgade P, Pouy A, Lyford-Pike P (2023). Doxycycline induced acute pancreatitis: case report. HPB.

[8] Shah N, Razzano A, Grendell J (2021). Doxycycline induced severe acute pancreatitis: a rare finding to a common medication. BMJ Case Rep.

[9] Goldin MR, Petry R, Goyal P (2024). Doxycycline-associated acute pancreatitis: a rare adverse effect of a commonly prescribed antibiotic. BMJ Case Rep.

[10] Banks PA, Bollen TL, Dervenis C (2013). Classification of acute pancreatitis-2012: revision of the Atlanta classification and definitions by international consensus. Gut.

[11] Torosis J, Vender R (1987). Tetracycline-induced pancreatitis. J Clin Gastroenterol.

[12] Kakes J, Cayley WE Jr, Sporleder J (2024). A case of doxycycline-induced pancreatitis. WMJ.

[13] Ksiądzyna D (2011). Drug-induced acute pancreatitis related to medications commonly used in gastroenterology. Eur J Intern Med.

[14] Evidencio. Naranjo (2024). Naranjo Adverse Drug Reaction Probability Scale. https://www.evidencio.com/models/show/661.

